# Letter to the Editor regarding Hubacek et al.’s report “Lack of an association between SNPs within the cholinergic receptor genes and smoking behavior in a Czech post-MONICA study”

**DOI:** 10.1590/1678-4685-GMB-2017-0214

**Published:** 2018-05-17

**Authors:** Ahmet Müderrisoglu, Melih Babaoglu

**Affiliations:** Hacettepe University Hacettepe University Department of Pharmacology Ankara Turkey Department of Pharmacology, Medical Faculty, Hacettepe University, Ankara, Turkey

**Figure 1:**
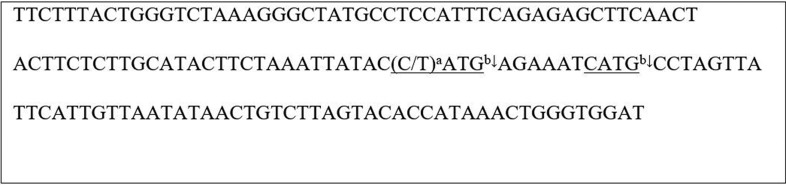
The genetic sequence of the PCR product of 146 bp for the analysis of *CHRNA3* rs578776 with the restriction sites for *Nla*III. (chromosome 15: 78595991-78596136, Accession Number: NM_000743.4). ^a^Single nucleotide polymorphism: (C/T); ^b^*Nla*III recognition sites underlined; ↓Restriction Points.

**Figure 2:**
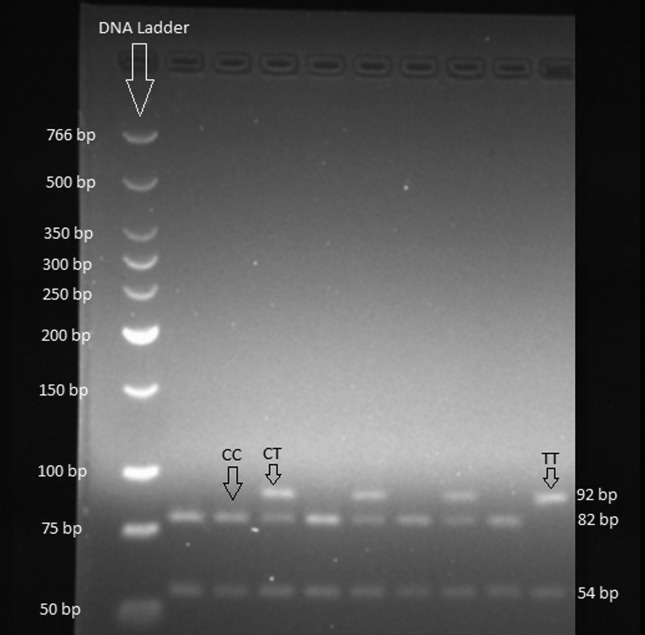
The actual restriction pattern of the PCR product for *CHRNA3* rs578776 by using *Nla*III.

In the article Hubacek *et al.* (2014) published in this journal, a genetic variant of *CHRNA3* was examined. We think that molecular sizes for the PCR product and the restriction fragments for the analysis of rs578776 were presented erroneously in Table 1. In this table, it said that the PCR product encompasses 167 base pairs (bp) and the restriction fragment sizes are 100 bp and 67 bp for the C allele when the PCR product was cut by the enzyme *Nla*III. However, the actual size of the PCR product should be 146 bp, and restriction fragments should be 82 bp, 54 bp and 10 bp for the C allele; and 92 bp and 54 bp for the T allele. In Figure 1 we describe here the PCR product and the restriction fragments predicted by using the University of California Santa Cruz (UCSC) Genome Browser *In Silico* PCR Tool with the primers 5’-TTC TTT ACT GGG TCT AAA GGG CTA TGC C-3’, 5’-ATC CAC CCA GTT TAT GGT GTA CTA AG-3’ and the restriction enzyme *Nla*III, as used in Table 1 of the article. We also performed an *in silico* restriction analysis using NEBcutter V2.0. Figure 2 demonstrates an actual example of the restriction pattern obtained in our laboratory.

Since other researchers who may wish to apply the methodology in the future may be misled by the erroneous information published, we would like to bring this error to attention for a possible correction.
